# Holistic view of biological nitrogen fixation and phosphorus mobilization in *Azotobacter chroococcum* NCIMB 8003

**DOI:** 10.3389/fmicb.2023.1129721

**Published:** 2023-02-08

**Authors:** Karolina A. Biełło, Carlos Lucena, Francisco J. López-Tenllado, Jesús Hidalgo-Carrillo, Gema Rodríguez-Caballero, Purificación Cabello, Lara P. Sáez, Víctor Luque-Almagro, María Dolores Roldán, Conrado Moreno-Vivián, Alfonso Olaya-Abril

**Affiliations:** ^1^Departamento de Bioquímica y Biología Molecular, Edificio Severo Ochoa, Campus de Rabanales, Universidad de Córdoba, Córdoba, Spain; ^2^Departamento de Botánica, Ecología y Fisiología Vegetal, Edificio Celestino Mutis, Campus de Rabanales, Universidad de Córdoba, Córdoba, Spain; ^3^Departamento de Química Orgánica, Instituto Universitario de Investigación en Química Fina y Nanoquímica (IUNAN), Universidad de Córdoba, Córdoba, Spain

**Keywords:** biological nitrogen fixation, phosphorus mobilization, nitrogenase, phosphatases, *Azotobacter chroococcum*, Proteomics

## Abstract

Nitrogen (N) and phosphorus (P) deficiencies are two of the most agronomic problems that cause significant decrease in crop yield and quality. N and P chemical fertilizers are widely used in current agriculture, causing environmental problems and increasing production costs. Therefore, the development of alternative strategies to reduce the use of chemical fertilizers while maintaining N and P inputs are being investigated. Although dinitrogen is an abundant gas in the atmosphere, it requires biological nitrogen fixation (BNF) to be transformed into ammonium, a nitrogen source assimilable by living organisms. This process is bioenergetically expensive and, therefore, highly regulated. Factors like availability of other essential elements, as phosphorus, strongly influence BNF. However, the molecular mechanisms of these interactions are unclear. In this work, a physiological characterization of BNF and phosphorus mobilization (PM) from an insoluble form (Ca_3_(PO_4_)_2_) in *Azotobacter chroococcum* NCIMB 8003 was carried out. These processes were analyzed by quantitative proteomics in order to detect their molecular requirements and interactions. BNF led to a metabolic change beyond the proteins strictly necessary to carry out the process, including the metabolism related to other elements, like phosphorus. Also, changes in cell mobility, heme group synthesis and oxidative stress responses were observed. This study also revealed two phosphatases that seem to have the main role in PM, an exopolyphosphatase and a non-specific alkaline phosphatase PhoX. When both BNF and PM processes take place simultaneously, the synthesis of nitrogenous bases and *L*-methionine were also affected. Thus, although the interdependence is still unknown, possible biotechnological applications of these processes should take into account the indicated factors.

## 1. Introduction

To ensure crop production, the synthesis of chemical fertilizers (*CF*) has greatly increased during last six decades, with nitrogen (N) fertilizers representing the highest costs for the total N + phosphorus (P) + potassium (K) fertilization. However, the use of *CF* has serious environmental and microbiological consequences in soils, water resources and air (Savci, 2012; Hu et al., 2015; Staley et al., 2018). It is assumed that the current agricultural systems have become highly nitrifying, largely due to the excessive use of N-based *CF*, thus leading the conversion of ammonium to nitrite and nitrate through aerobic biological actions (Hu et al., 2015). In fact, nitrification and denitrification, which are predominant in natural and agricultural soils, contribute up to 70% of the global emissions of nitrous oxide (Shcherback and Robertson, 2019). In addition, only 30% of the supplied N is incorporated into the plants, 10% is taken in livestock, and 5% is retained by humans as proteins (Cassman et al., 2002; Tilman et al., 2002; Bahadoran et al., 2015). Then, current crop systems are inefficient and cause serious environmental problems, such as increased emissions of nitrous oxide, and the accumulation of nitrate and nitrite in water resources and in the food chain. In this sense, the use of microorganisms as biofertilizers is considered an interesting alternative to increase or maintain crop production with a significant reduction in the use of *CF* (Singh et al., 2011; Olanrewaju et al., 2017). Bacterial biofertilizers are known as “Plant Growth Promoting Bacteria” (PGPB; Sathya et al., 2017) and can also act as biocontrol agents (Deketelaere et al., 2017). It has been described that PGPB, once applied to seeds, plants or soil, are capable of colonizing the rhizosphere or the internal spaces of target plants to promote their growth (Vessey, 2003) and even increase soil fertility (Singh et al., 2011; Bhardwaj et al., 2014; Nosrati et al., 2014). Thus, biofertilizers represent a more profitable green trend since they are an ecological, cheap and safe alternative to the use of *CF*, with a minimum expected environmental impact in an environmental galenics context (De Lorenzo, 2022). However, in order to design effective strategies with PGPB, it is essential to understand the molecular mechanisms involved in the improvement of crops, the relationships among different nutrients, and the impacts in the environment (Pereg and McMillan, 2015; Inostroza et al., 2017). Biological nitrogen fixation (BNF) and phosphorus mobilization (PM) are two of the main biochemical processes with high interest in the use of microorganisms as biofertilizers since N and P are the most common limiting elements (Vitousek et al., 2010).

Nitrogen is one of the most important nutrients for plant growth and crop production, and despite of molecular nitrogen (N_2_) is present in the atmosphere at the highest percentage (78%), only free-living and symbiotic N_2_-fixing organisms (diazotrophs) are able to transform the gaseous nitrogen into ammonium, as N-source. BNF is carried out through the oxygen-sensitive enzymatic complex known as nitrogenase, of which three types are known, containing molybdenum, iron or vanadium as cofactors (Rubio and Ludden, 2008; Cabello et al., 2019; Einsle and Rees, 2020). The predominant form of nitrogenase is the molybdenum (Mo)-associated form, that is encoded by the *nif* genes. This consists of a catalytic MoFe protein (dinitrogenase) and a Fe protein (dinitrogenase reductase), which binds ATP and participates in the maturation of the MoFe protein (Hoffman et al., 2014; Burén et al., 2020; Einsle and Rees, 2020). Previous studies of the BNF revealed that additional gene products are required for the assembly of the metal cofactors and maturation of the nitrogenase, like the NifB, NifE, NifN, NifM, NifS, NifU, and NifW proteins (Jacobson et al., 1989; Moreno-Vivián et al., 1989; Masepohl et al., 1993; Zheng et al., 1993; Yuvaniyama et al., 2000; Dos Santos et al., 2004; Johnson et al., 2005; Gavini et al., 2006; Curatti et al., 2007; Rubio and Ludden, 2008).

BNF is also closely related to essential elements, like carbon (Inomura et al., 2018) and phosphorus (Nosrati et al., 2014), which is the second main macronutrient that limits the growth of plants, affecting plant health and crop yield (Schachtman et al., 1998). In fact, P availability controls BNF (Aasfar et al., 2021). Unlike N, the supply of P depends mainly on the weathering of the original material (Walker and Syers, 1976; Vitousek et al., 2010) and P losses from soils cannot be replenished without external input, since plants can only take up free orthophosphate (Holdford and Cullis, 1985). However, this external contribution is often accompanied by undesirable additions of cadmium (Jafarnejadi et al., 2013; Molina-Roco et al., 2018), with a dangerous influence on human health. In this sense, microorganisms play an important role improving the mobilization and availability of P through degradation of the substrate (biological mineralization of P), and/or releasing extracellular enzymes (biochemical mineralization of P), complexes or compounds that dissolve and recycle minerals and organic phosphorus anions (McGill and Cole, 1981; Richardson and Hadobas, 1997; Tairo and Ndakidemi, 2013). The solubilization of inorganic P mainly occurs by the production of organic acids, which achieves solubilization by (i) lowering the pH, (ii) improving the chelation of the cations attached to P, (iii) competing with P for the sites of adsorption in the soil, or (iv) forming soluble complexes with metal ions (Ca^2+^, Al^3+^, Fe^3+^) associated with insoluble P. The solubilization of organic P, also called organic P mineralization, plays an imperative role in the phosphorus cycle of agricultural systems. The release of P from organic compounds by microbial enzymes may constitute up to 90% of the total P in soils (Khan et al., 2007) and, depending on the substrate, these enzymes can be classified into three groups: phosphatases (specific and non-specific), phytases, and phosphonates/C-P lyases (Wan et al., 2020). Effective PGPB have already been described to solubilize the precipitated and adsorbed forms of inorganic P (Schmalenberger and Fox, 2016). In addition, some microorganisms have efficient P absorption systems, such as the high affinity phosphate-specific transporter Pst and the low affinity inorganic phosphate transporter Pit (Willsky et al., 1973).

The “omics” techniques (genomics, transcriptomics, proteomics or metabolomics) provide holistic views that increase the global knowledge of the processes under study, such as the identification of metabolic responses and molecular mechanisms to nutrient stress (Lidbury et al., 2016, 2021; Murphy et al., 2022) or conditions like diazotrophic vs. non-diazotrophic conditions (Madeira et al., 2020; Nkongolo and Narendrula-Kotha, 2020). Since proteins are the final molecular effectors of gene functions, proteomics emerged as a necessary tool for the identification of proteins in a biological system, defining the proteome as the set of proteins expressed in a given organism/cell in response to certain conditions (Anderson and Anderson, 1998). However, a series of precautions must be taken when carrying out these studies, such as the batch effects (systematic nonbiological biases derived from sample preparation and measurement conditions; Zhou et al., 2019; Cuklina et al., 2020).

In this work, BNF and PM were analyzed by quantitative proteomics in *Azotobacter chroococcum* NCIMB 8003, a gram-negative bacterium capable of both BNF and PM (Overbeck and Malke, 1967; Chennapa et al., 2017) that belongs to a genus widely studied as biofertilizer in sustainable agriculture (van Oosten et al., 2018; Wakarera et al., 2022). Direct relationships between BNF and PM were already observed in this microorganism (Iswatran and Marwaha, 1981), like in other *Azotobacter* strains (Garg et al., 2001; Aasfar et al., 2021), but underlying mechanisms are still unknown. Therefore, this strain is a good model for the study of BNF and PM through holistic approaches. In this work we describe the global changes occurred at the protein level, which were functionally validated at gene transcription and enzyme activity levels. This knowledge will contribute to the identification, development, and optimization of effective biofertilizers for crops by biotechnological approaches.

## 2. Experimental procedures

### 2.1. Bacterial strains and growth conditions

*Azotobacter chroococcum* NCIMB 8003 was purchased from the Spanish Type Culture Collection (CECT, Valencia, University of Valencia). Cells were cultured at 30°C and 125 rpm into an orbital shaker in a nitrogen-free minimal liquid medium with the following composition: 110 mM glucose, 100 mM 3-(N-morpholino)propanesulfonic acid (MOPS) buffer pH 7.2, and metal traces (20 mg/L CaCl_2_, 10 mg/L Na_2_MoO_4_, 200 mg/L NaCl, 1.5 mg/L MnSO_4_, 0.3 mg/L CuSO_4_, 0.3 mg/L CoSO_4_, 0.1 mg/L H_3_BO_3_, 2.16 mg/L ZnSO_4_, 200 mg/L MgSO_4_ and 5 mg/L FeSO_4_). For non-diazotrophic conditions, 10 mM ammonium chloride was used since is the inorganic nitrogenous form that is incorporated into carbon skeletons. For non-phosphorus mobilization conditions, phosphate buffer containing 0.9 g/L K_2_HPO_4_ and 0.1 g/L KH_2_PO_4_ (pH 7.2) was used. Four growth conditions were assayed: control condition (C), with ammonium as N source and phosphate buffer as P source; diazotrophic condition (BNF), without ammonium and with phosphate buffer; phosphorus mobilization condition (PM), with ammonium and with 5 g/l tricalcium phosphate [Ca_3_(PO_4_)_2_] as P source; and diazotrophic and phosphorus mobilization condition, without ammonium and with 5 g/L tricalcium phosphate as P source. For this last condition, cells were harvested at two different times, at 24 and 72 h (FP and FPb respectively). For solid media, 15 g/L bacteriological agar was added prior sterilization by autoclave. The pH was monitored by using a pH meter. As inoculum, a previous culture grown under diazotrophic condition was centrifuged at 5,000 ×*g* for 10 min and resuspended on 0.85% NaCl. Initial absorbance at 600 nm was adjusted to 0.05 for all conditions.

### 2.2. Analytical determinations

All measurements were carried out in triplicate from three different biological replicates. Cell growth was determined by monitoring the absorbance of the bacterial cultures at 600 nm in a spectrophotometer (Spectronic™, Thermo Scientific) and by counting colony forming units (CFU) using the drop plate method (Herigstad et al., 2001). The concentration of ammonium was measured by the method described by Solórzano (1969), with minor modifications using a calibration plot previously elaborated with a 2 mM ammonium chloride stock solution. The concentration of soluble phosphorus was measured using the ascorbic acid method (Murphy and Riley, 1962), with modifications. Briefly, a reaction mix containing 12.5 mL 5 N sulfuric acid, 3.75 ml of 40 g/L ammonium molybdate, 7.5 mL 0.1 M ascorbic acid and 1 mg/mL antimony potassium tartrate was developed. In a 1.5 mL tube, 800 μL of suitably diluted supernatant samples and 200 μL of the reaction mixture were mixed, and after 8 min incubation at room temperature, the absorbance at 882 nm was determined. Soluble P concentration was calculated using a calibration plot previously elaborated with a stock solution of 25 μg/mL KH_2_PO_4_. The concentration of proteins was measured by using the method of Bradford (1976), based in the binding of protein molecules to Coomassie dye under acidic conditions that results in a color change from brown to blue. After 5 min incubation at room temperature in darkness, the absorbance at 595 nm was determined. Protein concentration was calculated by using a calibration plot previously elaborated with a stock solution of 200 μg/mL bovine serum albumin.

### 2.3. Proteome analysis

*Azotobacter chroococcum* NCIMB 8003 cells were grown aerobically in four growth conditions [control, C; diazotrophic, BNF; phosphorus mobilization from an insoluble form (Ca_3_(PO_4_)_2_), PM; and diazotrophic and phosphorus mobilization, FP], in triplicate. Since a significant release of ammonium into the culture medium was observed in FP, samples were collected at 24 and 72 h (FP and FPb, respectively). Ammonium-grown cells were harvested when ammonium consumption reached ~70%, and for diazotrophic conditions, cells were harvested when a similar number of CFU was reached. Then, all cultures were placed 2 min on a surface, in order to allow the insoluble P to settle in those samples that contained it, and 25 mL per replica were centrifugated at 12,000 rpm for 15 min. Samples for LC-MS/MS proteomic analysis were resuspended in lysis buffer (50 mM Tris–HCl, pH 8.0) containing 4% 3-((3-cholamidopropyl) dimethylammonio)-1-propanesulfonate (CHAPS) and 8 M urea and disrupted by cavitation with ultrasounds (6 pulses for 20 s at 25 W in a Bandelin Sonoplus HD2070 equipment). Then, samples were cleaned with the 2-D Clean-UP Kit (GE Healthcare, Little Chalfont, United Kingdom) and resuspended in 200 μL lysis buffer. Protein concentration was estimated and 10 μL of the protein solution were digested with 2 μg trypsin overnight at 37°C without agitation. Finally, same amount of iRT (Biognosys) were added to all samples and 1 μg total protein was analyzed at the Research Support Central Service (SCAI), University of Cordoba, as previously described (Olaya-Abril et al., 2021; Pérez et al., 2021). Then, MS2 spectra were searched by using MaxQuant software v2.2.0.0, with Andromeda as search engine against a database of *A. chroococcum* NCIMB 8003 deposited in Uniprot (UP000068210). The search and quantification parameters used for the proteomic analysis are shown in Supplementary Table S1. Data were analyzed by using Perseus software (1.6.12.1)^1^ and a GO enrichment analysis was carried out by using the Comparative GO application (Fruzangohar et al., 2013). When required, the PSORTb 3.0 algorithm (Yu et al., 2010) was used to analyze the possible subcellular localization of proteins of interest. Data were deposited to the ProteomeXchange Consortium^2^ *via* the PRIDE partner repository with the dataset identifier PXD034112.

### 2.4. Enzyme activities

Phosphatase activities were determined from 25 mL, per replicate, of the samples previously decanted for 2 min. Then, cell extracts were obtained by sonication (3 pulses for 7 s at 20 W). For both acid and alkaline phosphatases, an “stopped-time” assay based upon the hydrolysis of *p*-nitrophenyl-phosphate (pNPP) was used, and an extinction coefficient at 410 nm (ε410) of 18.2 mM^−1^ cm^−1^ for *p*-nitrophenol (pNP) was considered. In the case of acid phosphatase assay, the procedure of Campbell et al. (1978) was carried out, with minor modifications. In brief, a mix containing 500 μL acetate buffer (pH 5.0, 200 mM) and 2 mM substrate (pNPP) was incubated with up to 500 μL sample (500 μL supernatant for extracellular phosphatase activity and 100 μL crude extract plus 400 μL acetate buffer for intracellular phosphatase activity) at room temperature for 10 min. For alkaline phosphatase activity assay, 300 μL assay buffer, consisting of 100 mM glycine (pH 10.4), 1 mM MgCl_2_ and 1 mM ZnCl_2_, were mixed with 200 μL 0.3 mM substrate (pNPP) prepared in assay buffer and 500 μL sample (Wachstein, 1946). Both phosphatase assays were stopped by adding 200 μL 3 M NaOH after 10 min incubation at room temperature. Finally, absorbance was measured at 410 nm in a spectrophotometer. Protein quantification was determined by the Bradford assay (Bradford, 1976).

Nitrogenase activity was measured by using the acetylene reduction assay (Hardy et al., 1968), which uses acetylene as an alternative substrate that is reduced to ethylene, determined by gas chromatography. Briefly, 5 mL cell cultures were transferred to tubes with rubber stoppers and purged with argon for 30 min until gas atmosphere was replaced. Then, cells were incubated at 30°C for 15 min followed by an injection of 1 mL of acetylene. For each sample, 0.4 mL headspace was analyzed by gas chromatography (Agilent Technologies chromatograph using a Supelco Carboxen™ 1,010 PLOT fused silica capillary column, 30 m long, 0.32 mm ID) and ethylene production was calculated.

### 2.5. RNA quantification by qRT-PCR

RNA isolation, cDNA synthesis and cDNA quantitation were carried out from cells grown under the different experimental conditions, as previously described (Olaya-Abril et al., 2018, 2019), in triplicate, from 25 mL of the same samples used for the proteomic analysis. Gene-specific primers were designed using the Oligo 7.0 software (Supplementary Table S2). Data were normalized to the *rpoB* housekeeping gene.

### 2.6. Statistical analysis

All statistical analysis were carried out using the Perseus software, with data acquired from three biological replicates. For proteomic data, first, an exploratory analysis was carried out by counting all proteins per replicate and unique proteins per sample and global analysis (Supplementary Dataset S1). Then, relative label free quantification (LFQ) intensities values were transformed to log_2_ LFQ intensity and a cluster analysis and a principal component analysis (PCA) were developed by using default parameters. In the case of PCA, missing values were replaced by values from a normal distribution. For differential expression analysis, a *t*-test analysis with the Benjamini-Hochberg correction method were applied on the original log_2_ LFQ intensities and differential expressed proteins were defined as those with an adjusted *p*-value ≤ 0.05 and a fold change (FC) ≥ 2. Proteins tagged as “exclusively expressed” were identified in at least two of the three replicates of one condition and in none of the replicates of the other condition. For qRT-PCR analysis, normalized data were analyzed by a *t*-test on the relative gene expression, as previously described, and relative fold gene expression was calculated by the ΔΔCt method (Olaya-Abril et al., 2022).

## 3. Results and discussion

### 3.1. Growth of *Azotobacter chroococcum* NCIMB 8003 under N_2_ fixation and P mobilization conditions

**Figure 1:**
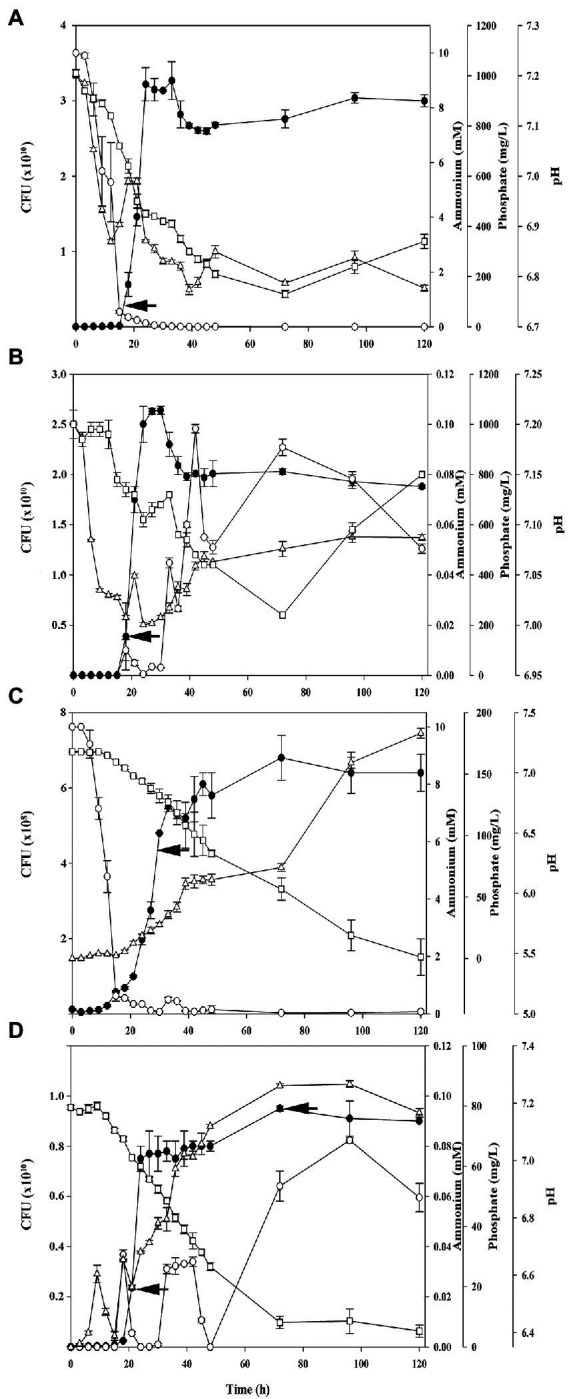
Growth curves and extracellular ammonium (mM), phosphorus (mg/L) and pH of *Azotobacter chroococcum* NCIMB 8003 cultures in the different conditions used: **(A)** Control, with 10 mM ammonium chloride and soluble phosphorus, **(B)** Diazotrophic condition (BNF), with soluble phosphorus and without ammonium chloride, **(C)** Phosphorus mobilization condition (PM), with 10 mM ammonium chloride and with tricalcium phosphate as sole phosphorus source, and **(D)**, Diazotrophic and phosphorus mobilization condition (FP), without ammonium chloride and with tricalcium phosphate. For each condition, bacterial growth is shown as CFU counting (black circles), extracellular ammonium (circles), extracellular soluble phosphorus (triangles) and extracellular pH (squares). Cells were precultured in nitrogen-free minimal medium with 110 mM glucose as carbon source for 24 h. Then, cells were harvested by centrifugation, washed in 0.85% NaCl and inoculated to an initial optical density at A600 of 0.05. The arrows show the collection times of the different conditions for further analysis.

*Azotobacter chroococcum* NCIMB 8003 is a gram-negative bacterium able to carry out both biological nitrogen fixation (BNF) and phosphorus mobilization (PM; Overbeck and Malke, 1967; Chennapa et al., 2017). Although the relationship and dependence between both processes is known in *Azotobacter* species (Aasfar et al., 2021), they have not been studied simultaneously in the same organism using a holistic approach like quantitative proteomics. Nevertheless, before carrying out the proteomic analysis, bacterial growth was characterized over time by counting CFU and by measuring the amount of ammonium and phosphorus in the culture supernatants. In addition, pH of the media was monitored (Figure 1). In the control condition (C, culture media with ammonium chloride and phosphate buffer) the best growth rates were obtained at ~30 h, when the N-source was consumed. The pH decreased from 7.21 to 6.87 at the end of the experiment and phosphate remained in excess (Figure 1A). In the nitrogen fixation condition (BNF, culture media without ammonium plus phosphate buffer) the maximal growth slightly decreased with respect to C condition, and transient oscillations of NH_4_^+^ in the supernatant were observed, with the highest detection at 42 h, when ~100 ± 1.7 μM ammonium was measured. Phosphate was kept in excess and pH decreased moderately, ranging between 7.20 and 7.01 (Figure 1B). In the phosphorus mobilization condition (PM, with ammonium chloride as N-source and tricalcium phosphate as sole P-source) growth was much lower during the incubation time, which would explain the detection of traces of ammonium even at 120 h. However, the presence of phosphate in the supernatant increased throughout the time (Figure 1C). The drop of pH was very pronounced (from 7.19 to 5.47), thus requiring the addition of MOPS to the culture medium, since without this buffer addition cell viability fell rapidly after 24 h (Supplementary Figure S1). This is consistent with the requirement of phosphate buffer in the media described by Thompson and Smith (1931). Then, before its incorporation for subsequent experiments, it was ensured that MOPS was not used as a N-source by incubating *A. chroococcum* in an inert atmosphere (data not shown). Finally, a physiological characterization was also carried out on *A. chroococcum* under nitrogen fixation and phosphorus mobilization conditions (FP, without ammonium and without phosphate buffer). In this case, the growth ratio was improved with respect to PM, but it was still lower than in the BNF condition. The pH dropped from 7.19 to 6.41, showing also intermediate levels between BNF and PM (Figure 1D). Regarding the presence of ammonium in the extracellular medium, transient oscillations were also observed in this FP condition, with peaks of about 30 μM during the exponential growth and the highest values reaching 60–80 μM at the end of the experiment (72–120 h). As transient oscillations of ammonium were also observed in the BNF condition, in which phosphorus was in excess, it can be assumed that phosphorus is not directly related to the release of ammonium during diazotrophic growth.

### 3.2. *Azotobacter chroococcum* NCIMB 8003 proteome under N_2_ and P mobilization conditions

*Azotobacter chroococcum* NCIMB 8003 was grown in the four previously described conditions (C, BNF, PM, and FP), in triplicate. Subsequently, 25 mL of each replica were taken for subsequent analyses: proteomic, qRT-PCR and enzymatic activities. The sampling time (showed with black arrows in Figure 1) was selected considering that the process under study (nitrogen fixation/phosphorus mobilization) was being carried out, but also: (1) the number of cells, to guarantee the sufficient extraction of proteins or RNA according to our previous experience (Olaya-Abril et al., 2018, 2019, 2020, 2021), and considering the lower yields in the PM condition; (2) the presence of ammonium in the non-diazotrophic conditions to exclude N-starvation response; and (3) the detection of phosphorus in the extracellular medium, especially under conditions of phosphorus mobilization. Therefore, in the FP condition two points were taken; the first (FP, 24 h) corresponds to a BNF and PM condition, and the second (FPb, 72 h) to a PM condition in which ammonium is being released. Number of CFU, ammonium and P concentration in the media, and pH at sampling time are shown in Supplementary Table S3. Then, cells were processed to perform a LC-MS/MS analysis.

**Figure 2:**
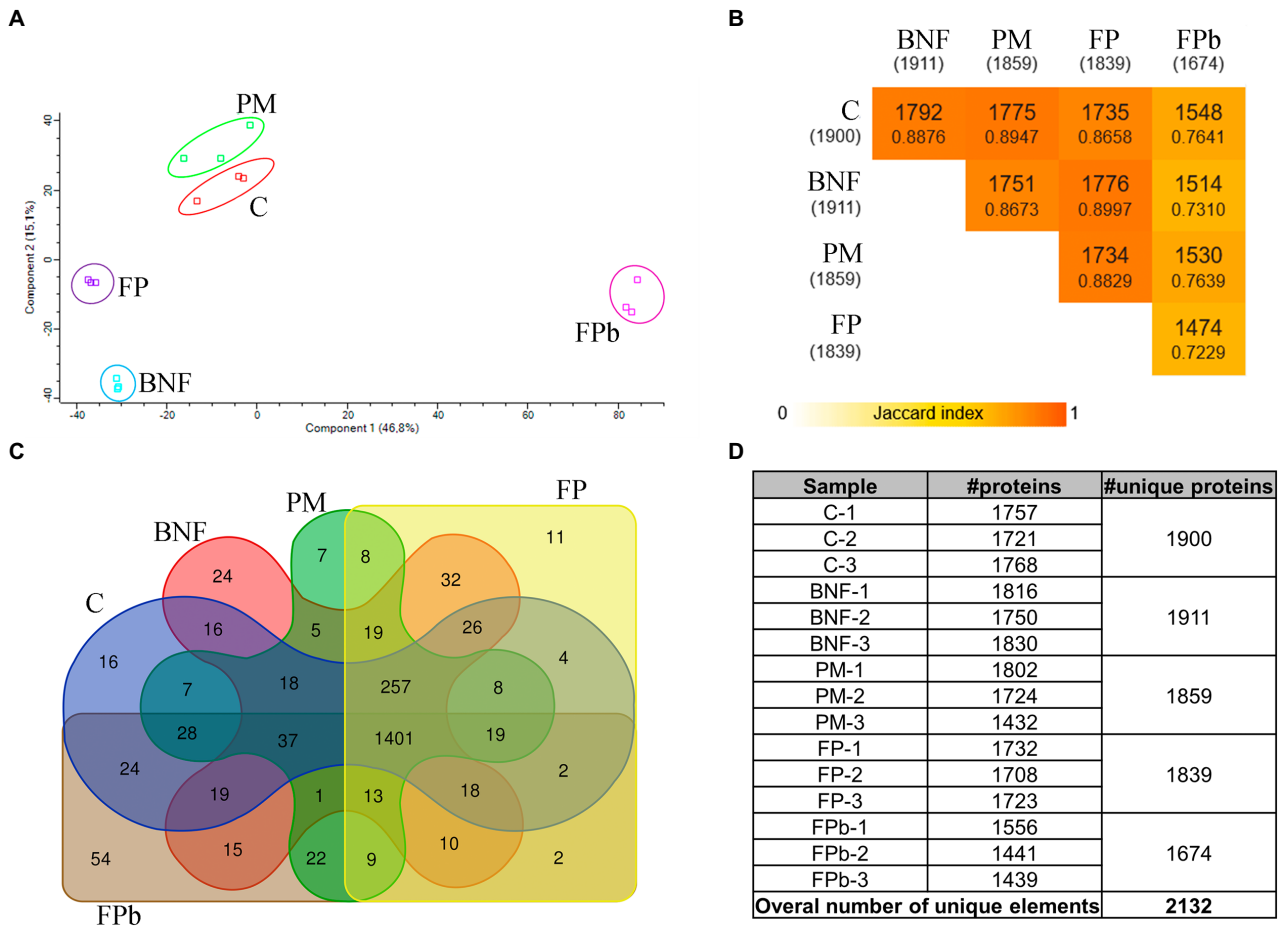
Exploratory proteome analysis. **(A)** Principal component analysis, **(B)** Jaccard index, **(C)** Venn diagram, **(D)** Summary table of the number of proteins identified per replicate, condition and in total. C, control condition; BNF, biological nitrogen fixation condition; PM, phosphorus mobilization condition; FP, biological nitrogen condition and phosphorous condition (21 h); FPb, biological nitrogen condition and phosphorous condition (72 h).

A total of 2,132 unique proteins were identified from 4,358 structural genes present in the whole genome of *A. chroococcum* NCIMB 8003 (Supplementary Dataset S2; Supplementary Figure S2). A principal component analysis (PCA), whose two first components together account for 61.9% of the total variance, showed that the three replicates of the 5 experimental conditions were grouped independently, with C and PM being close, FP and BNF being more separated, and FPb being located further away, along the first component (Figure 2). This was consistent with the hierarchical clustering analysis (Supplementary Figure S2). Subsequently, the quantitative analysis (Supplementary Dataset S3) was performed, and for a first approximation, an enrichment analysis of GO categories was carried out (Supplementary Figure S3). When BNF and C were compared, GO categories “protoporphyrinogen IX biosynthetic process,” “nitrogen fixation,” and “*de novo L*-methionine biosynthetic process” were enriched among proteins induced in BNF. In PM vs. C, a GO terms enrichment was observed in “phosphate ion transport” among proteins induced in PM, whereas “iron–sulfur cluster assembly” category was found in proteins induced in C condition. “Nitrogen fixation” and “*de novo L*-methionine biosynthetic process” were enriched again among proteins induced in FP compared to C, thus indicating that these GO categories, among others, are related to BNF. Finally, when FPb was compared to C, “amino acid transport,” “response to oxidative stress,” “molybdate ion transport,” “cellular iron ion homeostasis,” and “iron ion transport” were enriched among proteins induced in FPb, whereas proteins induced in C showed GO enrichments in “acetyl-CoA biosynthetic process,” “acetyl-CoA metabolic process,” “acetate metabolic process,” and “glyoxylate cycle” (Supplementary Figure S3).

**Figure 3:**
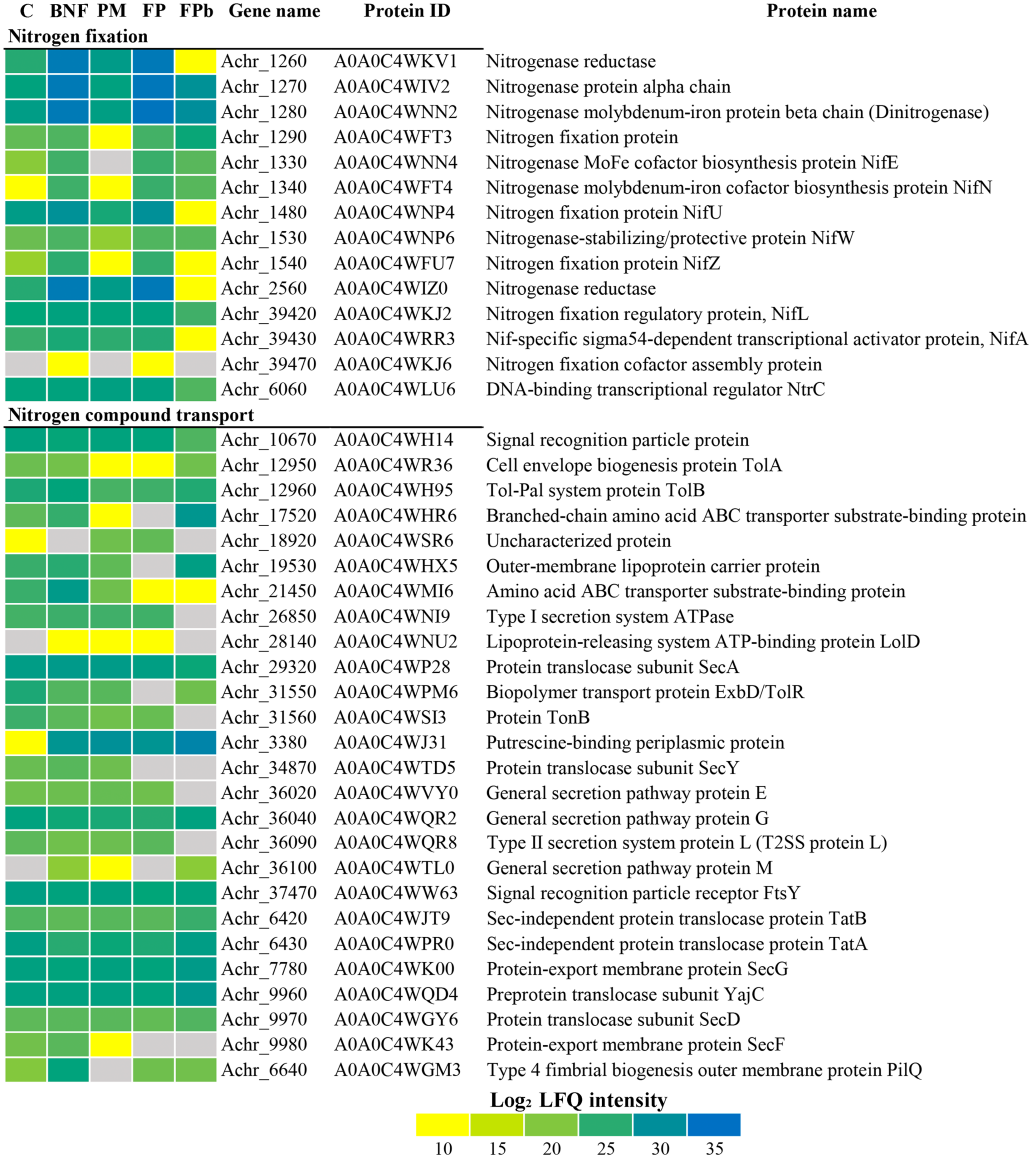
Differential expression of proteins related to nitrogen metabolism differentially expressed. Data are shown as heatmap of Log_2_ normalized peptide intensity. C, control condition; BNF, biological nitrogen fixation condition; PM, phosphorus mobilization condition; FP, biological nitrogen condition and phosphorous condition (21 h); FPb, biological nitrogen condition and phosphorous condition (72 h).

**Figure 4:**
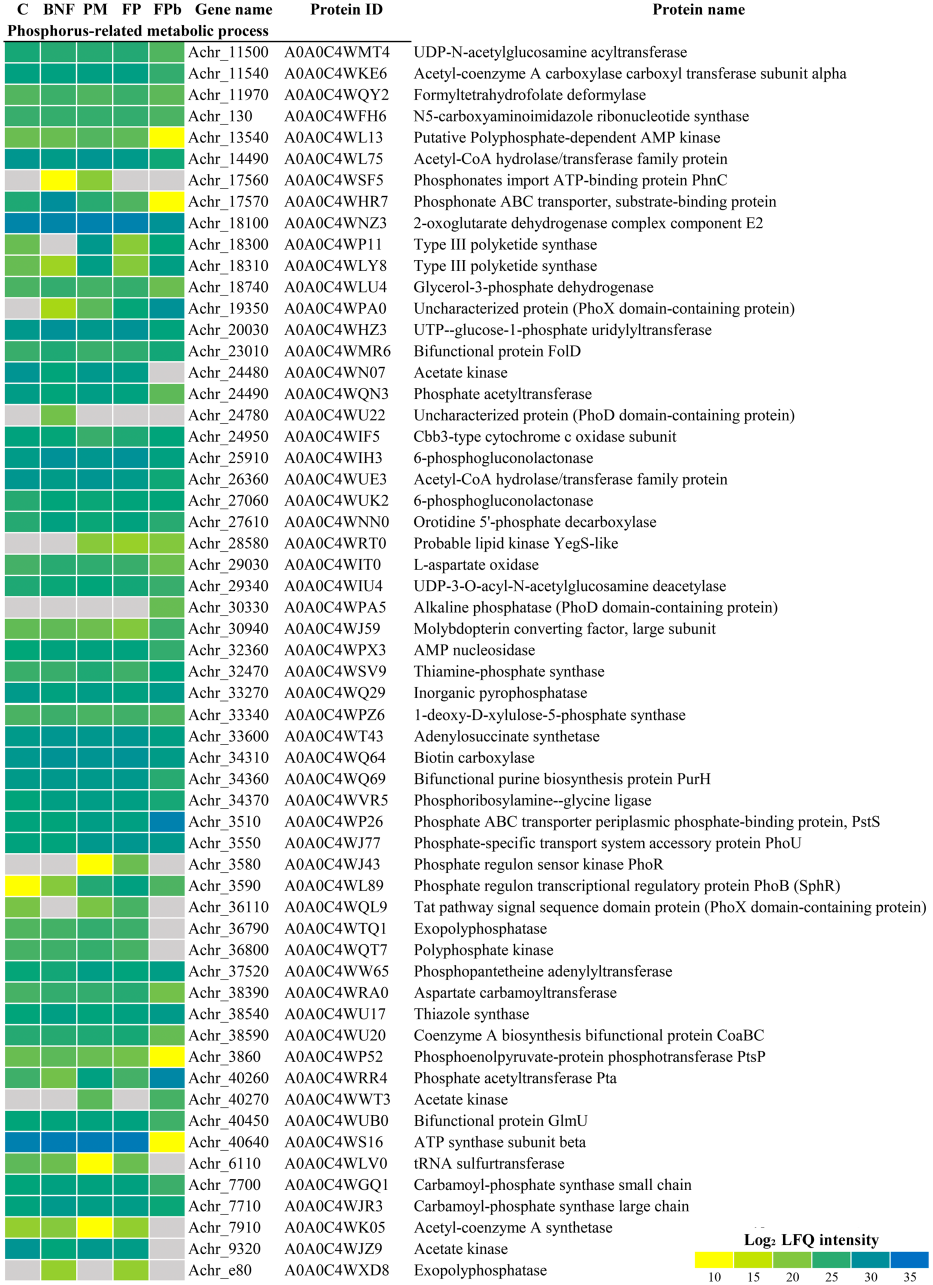
Differential expression of proteins related to phosphorus metabolism. Data are shown as heatmap of Log_2_ normalized peptide intensity. C, control condition; BNF, biological nitrogen fixation condition; PM, phosphorus mobilization condition; FP, biological nitrogen condition and phosphorous condition (21 h); FPb, biological nitrogen condition and phosphorous condition (72 h).

**Figure 5:**
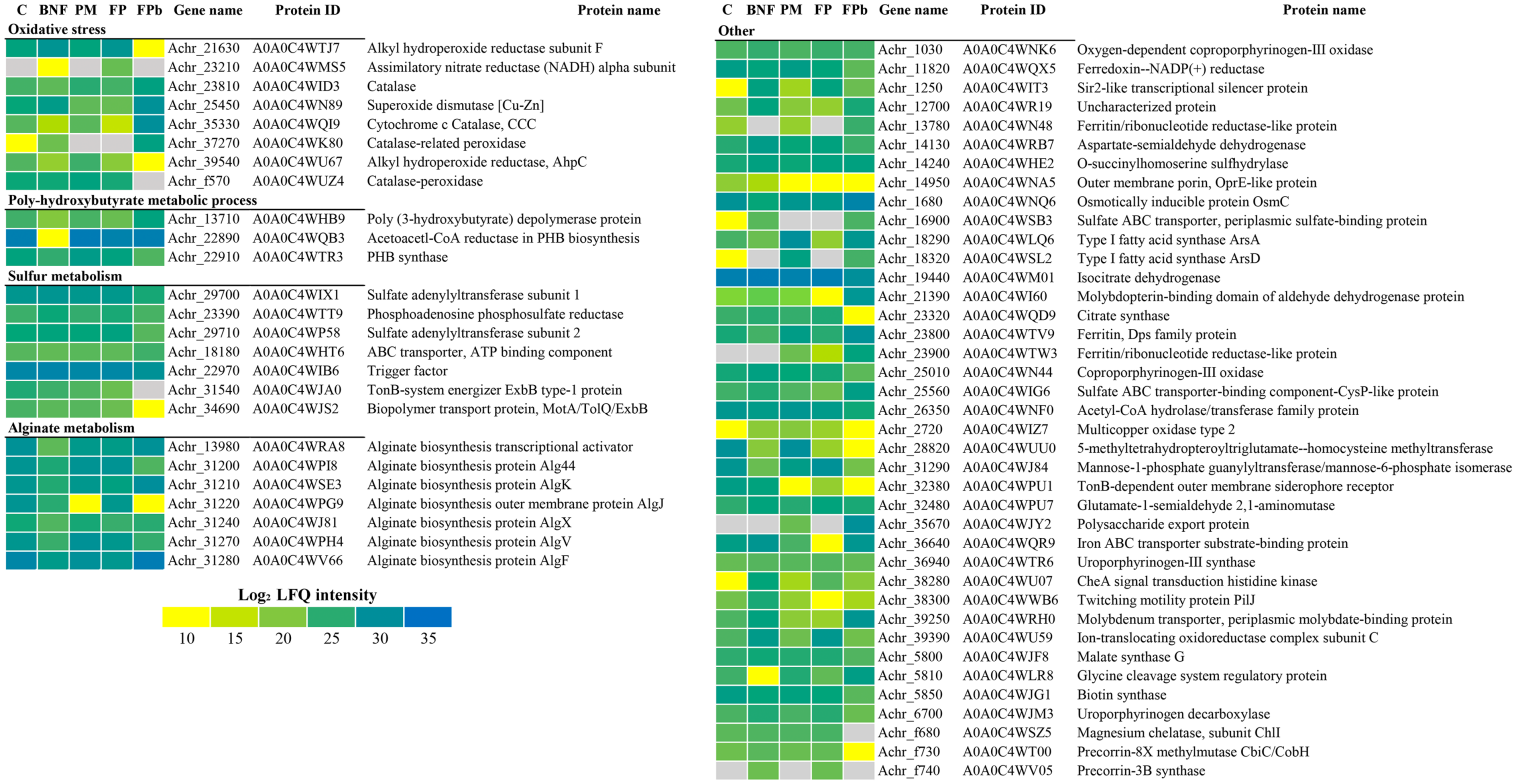
Differential expression of some proteins related to oxidative stress, metabolism of poly-hydroxybutyrate, sulfur, alginate and other processes. Data are shown as heatmap of Log_2_ normalized peptide intensity. C, control condition; BNF, biological nitrogen fixation condition; PM, phosphorus mobilization condition; FP, biological nitrogen condition and phosphorous condition (21 h); FPb, biological nitrogen condition and phosphorous condition (72 h).

Proteins related to N_2_ fixation and N metabolism were found differentially expressed (Figure 3). A general decrease in the levels of Nif proteins can be observed in the FPb condition compared to BNF and FP, indicating that the biological nitrogen fixation was negatively affected. In fact, the levels of the transcriptional activator NifA (A0A0C4WRR3, Achr_39430), the nitrogenase reductases NifH and VnfH (A0A0C4WKV1, Achr_1260 and A0A0C4WIZ0, Achr_2560, respectively), and the proteins NifU (A0A0C4WNP4, Achr_1480) and NifZ (A0A0C4WFU7, Achr_1540), that are required for the maturation of nitrogenase (Johnson et al., 2005), showed a marked decrease in the FPb condition. In the rest of the conditions, the expected expression pattern was observed with respect to Nif proteins. For some proteins involved in transport processes related to nitrogen metabolism, up-representation in FPb was observed, such as the putrescine-binding periplasmic protein (A0A0C4WJ31, Achr_3380), the general secretion pathway protein G (A0A0C4WQR2, Achr_36040), and the branched-chain amino acid ABC transport substrate-binding protein (A0AOO4WHR6, Achr_17520). Regarding proteins probably involved in phosphorus metabolism (Figure 4), a high induction of the PhoX domain-containing protein A0A0C4WPA0 (Achr_19350) was observed. This is an uncharacterized protein (according to KEGG annotation) or a Tat pathway signal protein (according to Uniprot) with unknown location according to PsortB. However, a PhoX domain is detected by InterPro and Pfam (residues 75-626), being recognized as a member of the alkaline phosphatase PhoX family and its location was experimentally confirmed as outer membrane anchored lipoprotein (Putker et al., 2013; Shropshire et al., 2021). This protein was not present in the control condition C, and an increased expression was found in the other experimental conditions, especially in FPb (FC 4597 in the comparison FPb vs. BNF, 107 in PFb vs. PM and 11.7 in FPb vs. FP). This data correlate with those previously described about the main role of the alkaline phosphatase PhoX in the phosphorus solubilization activity in proteobacteria (Monds et al., 2006; Sebastian and Ammerman, 2009; Lidbury et al., 2016). Protein encoded by Achr_36110 (A0A0C4WQL9) is another protein annotated as uncharacterized/tat related whose induction is also significant in FP vs. C (FC 8.46), FP vs. BNF and FP vs. PM (FC 9), but it was not detected in FPb. On the other hand, it was especially relevant the decrease in the FPb condition of the ATP synthase β subunit (A0A0C4WS16, Achr_40640), the phosphonate ABC transporter (A0A0C4WHR7, Achr_17570), the polyphosphate-dependent AMP kinase (A0A0C4WHL13, Achr_13540) and the phosphoenolpyruvate-protein phosphotransferase PtsP (A0A0C4WP52, Achr_3860). Likewise, the alkaline phosphatase-PhoD domain containing protein (A0A0C4WPA5, Achr_30330), the phosphate ABC transporter component PstS (A0A0C4WP26, Achr_3510) and the phosphate acetyltransferase Pta (A0A0C4WRR4, Achr_40260) showed an over-representation in the FPb condition. The PhoD domain-containing protein (A0A0C4WU22) encoded by the Achr_25780 gene was detected only in the BNF condition. A probable lipid kinase YegS-like (A0A0C4WRT0, Achr_28580) and the ferritin/ribonucleotide reductase-like protein (A0A0C4WTW3, Achr_23900) were found exclusively when phosphorus mobilization was operative (PM, FP and FPb), while the PhoR protein (A0A0C4WJ43, Achr_3580) was found exclusively in PM and FP. BNF and PM had effect on the metabolism of *A. chroococcum* beyond the metabolism of N and P (Figure 5). For example, some oxidative stress-related proteins were identified as differentially expressed. Thus, catalase (A0A0C4WID3, Achr_23810), superoxide dismutase (A0A0C4WN89, Achr_25450), cytochrome *c* catalase (A0A0C4WQI9, Achr_35330) and catalase-related peroxidase (A0A0C4WK80, Achr_37270) were found up-represented in FPb. Nevertheless, the alkyl hydroperoxide reductase subunit F (A0A0C4WTJ7, Achr_21630) was related to biological nitrogen fixation (BNF and FP conditions), and AhpC (A0A0C4WU67, Achr_39540) was found mostly in C and PM. The type I fatty acid synthase ArsD (A0A0C4WSL2, Achr_18320) and the polysaccharide export protein (A0A0C4WJY2, Achr_35670) showed an induction when the phosphorus mobilization process was triggered. Poly-hydroxybutyrate (PHB) and alginate biosynthesis-related proteins decreased in BNF compared to C. Acetoacetyl-CoA reductase (A0A0C4WQB3, Achr_22890) and PHB synthase (A0A0C4WTR3, Achr_22910), involved in PHB biosynthesis, and Alg44, Algk, AlgJ, AlgX, AlgV, and AlgF proteins, which are required for alginate biosynthesis, decrease their levels in BNF under our experimental conditions, which agrees with the relationship between alginate production and culture conditions described by Nosrati et al. (2012). However, the levels of the transcriptional activator GacA (A0A0C4WT38, Achr_20080) increased significantly in BNF (3-fold) and FP (2.8-fold). This regulator is a component of the GacS/A system involved in the control of alginate synthesis through transcriptional activation of a family of small RNAs (Núñez et al., 2022).

### 3.3. Enzyme activities and alginate production

**Figure 6:**
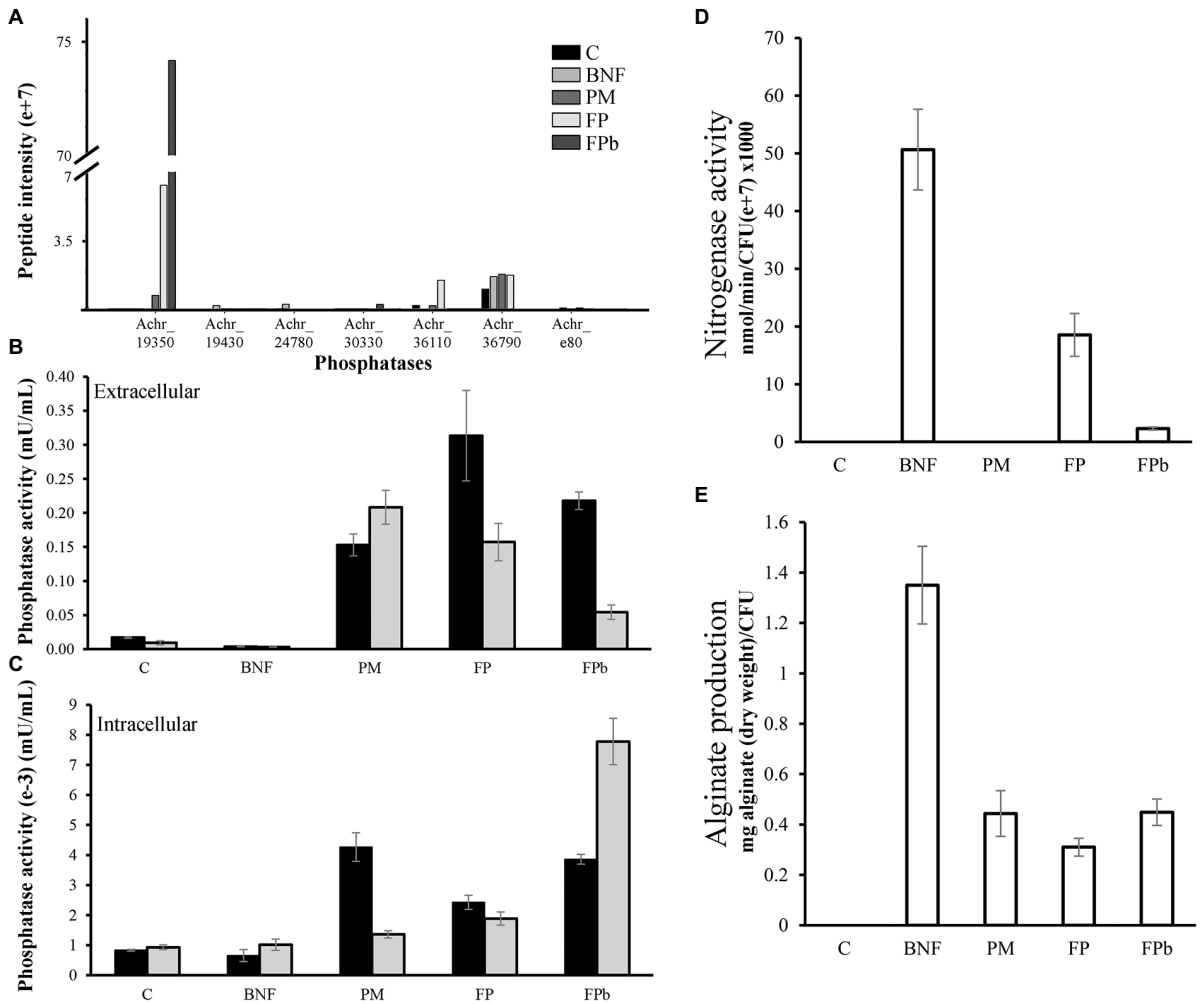
Phosphatase activities under the different experimental conditions. **(A)** Peptide intensities of the main phosphatases identified by proteomics. Extracellular **(B)** and intracellular **(C)** acid (black bars) and alkaline (gray bars) phosphatase activities. Achr_19430 (A0A0C4WHX1), phosphatase NudJ; Achr_30330 (A0A0C4WPA5), alkaline phosphatase; Achr_e80 (A0A0C4WXD8), Achr_36790 (A0A0C4WTQ1), exopolyphosphatase. **(D)** Nitrogenase activity measured by acetylene reduction assay. **(E)** Alginate production (mg dry weight/CFU) determined by gravimetric method. C, control condition; BNF, biological nitrogen fixation condition; PM, phosphorus mobilization condition; FP, biological nitrogen condition and phosphorous condition (21 h); FPb, biological nitrogen condition and phosphorous condition (72 h).

The genome of *A. chroococcum* NCIMB 8003 codes for several phosphatases that could contribute to the solubilization of phosphorus. Among them, there are an acid phosphatase (A0A0C4WQ17, Achr_33070), a protein with 11 transmembrane domains, two extracellular alkaline phosphatases (A0A0C4WJR7, Achr_6220; A0A0C4WPA5, Achr_30330), two cytosolic exopolyphosphatases (A0A0C4WTQ1, Achr_36790; A0A0C4WXD8, Achr_e80), and the phosphatase NudJ (A0A0C4WHX1, Achr_19430), which is also located in the cytosol. In addition, there are other possible phosphatases that are not annotated with this function, such as the previously mentioned PhoX domain-containing proteins A0A0C4WPA0 (Achr_19350) and A0A0C4WQL9 (Achr_36110) and the PhoD domain contain protein A0A0C4WU22 (Achr_24780). The alkaline phosphatase PhoX Achr_19350 (A0A0C4WPA0) was the most abundant in the FP conditions and the exopolyphosphatase Achr_36790 (A0A0C4WTQ1) was the most abundant in C, BNF, and PM conditions (Figure 6A). The transmembrane acid phosphatase was not detected in any of the conditions tested, probably due to the limitations of the technique (Bogdanow et al., 2016). This could also explain why the phosphatase activities assayed in the extra and intracellular media (Figures 6B,C) did not correlate well with the abundance of predicted as extracellular and intracellular proteins. On the other hand, the subcellular localization of the PhoX domain containing-proteins is unknown, and algorithms such as PsortB do not show conclusive results regarding their prediction of subcellular localization. In addition, exopolyphosphatase activities are usually assayed with buffers at similar pH than alkaline phosphatases (Akiyama et al., 1993). However, these activities have relationship with the extracellular pH of the culture, where the low pH increases acid phosphatase activities in the extracellular media. At the intracellular level, the alkaline phosphatase activity predominated in FPb while in PM the acid activity was predominant. Considering that insoluble phosphate is found outside the cell, the release of membrane vesicles (Olaya-Abril et al., 2014) enriched in acid phosphatase could be a mechanism involved in phosphorus mobilization, although additional studies would be needed to test this hypothesis. Regarding to the BNF process, the highest fixation rates were found in the BNF condition, not being detected in C or PM and being significantly lower in FP (Figure 6D). In FPb only a residual nitrogenase activity was detected, which agrees with the proteomic data. Finally, concerning to the production of alginate, it was not detected in the control condition, but quantified with the highest values in the BNF condition, and with similar intermediate values in the PM, FP, and FPb conditions (Figure 6E). These data do not correlate with those observed by proteomics for BNF and C. However, the transcriptional activator GacA (A0A0C4WT38, Achr_20080), was up-represented in the BNF condition compared to C. A high synthesis of alginate with a low amount of protein could involve post-translational regulation mechanisms, not addressed in this work. However, this difference could also reflect an imbalance between the synthesis and degradation of the proteins involved in this process. In this case, at the sample collection time, the proteins in the BNF condition could have a higher rate of degradation (with the alginate already produced), while in the control condition, they would be expressed for the subsequent synthesis of alginate. In any case, more studies are required to validate any of the hypotheses made.

### 3.4. Functional validation by qRT-PCR analysis

**Figure 7:**
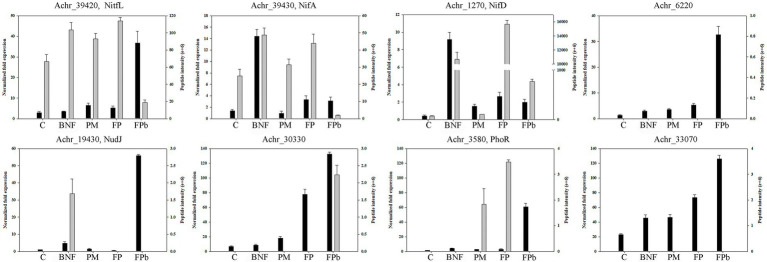
Transcriptional analysis of some genes coding for relevant proteins related to biological nitrogen fixation and phosphorus mobilization, and comparison with the peptide intensities obtained by proteomics. RNA levels (black bars) and peptide intensity (gray bars) are shown. Achr_39420 (A0A0C4WKJ2), NifL; Achr_39430 (A0A0C4WRR3), NifA; Achr_1270 (A0A0C4WIV2), NifD; Achr_6220 (A0A0C4WJR7), alkaline phosphatase; Achr_19430 (A0A0C4WHX1) phosphatase NudJ; Achr_30330 (A0A0C4WPA5), alkaline phosphatase; Achr_3580 (A0A0C4WJ43), phosphate regulon sensor kinase PhoR; Achr_33070 (A0A0C4WQ17) acid phosphatase/vanadium-dependent haloperoxidase superfamily. C, control condition; BNF, biological nitrogen fixation condition; PM, phosphorus mobilization condition; FP, biological nitrogen condition and phosphorous condition (21 h); FPb, biological nitrogen condition and phosphorous condition (72 h).

To overcome possible limitations of the proteomic approach, data were validated and completed by qRT-PCR (Figure 7; Supplementary Figure S4). Great differences were not observed in the levels of *nifL* mRNA in C, BNF, PM and FP, but *nifL* expression was increased in FPb, which could explain the decrease of nitrogen fixation, possibly as a consequence of a metabolic adjustment to the need for phosphate when carbon is in excess. The *nifA* transcript levels increased significantly in BNF, but did not change between FP and FPb, so that the significant decrease observed at the protein level could be due to posttranscriptional regulation of NifA. Likewise, expression of the *nifD* gene was higher in BNF and FP than in PM and FPb. Otherwise, although the alkaline phosphatase Achr_6220 and the acid phosphatase Achr_33070 were not detected by proteomics, their RNA levels were found significantly increased in FPb, as well as the RNA levels of the phosphatase NudJ (Achr_19430) and the alkaline phosphatase Achr_30330, which were detected at the proteomic level in the conditions BNF or FPb, respectively. Expression of Achr_3580 (*phoR*) also increased at the transcript level in the condition FPb, although the sensor-kinase PhoR protein was only detected in the proteomic analysis in PM and FP. The fact that extracellular acid phosphatase levels were higher in FPb than in PM, despite the pH in PM was lower, can be also explained by the reciprocal control relationship between nitrogen fixation and phosphorus mobilization (Nosrati et al., 2014) or by the existence of other phosphatases not yet identified as such, as in the case of the PhoX domain-containing protein A0A0C4WPA0 (Achr_19350).

### 3.5. Global vision of BNF and PM in *Azotobacter chroococcum* NCIMB 8003

The development of biotechnological applications of biological nitrogen fixation is one of the great goals for the future, as it would allow the use of microorganisms as efficient biofertilizers (Devi et al., 2022) or the development of transgenic plants capable of fixing atmospheric nitrogen (Burén and Rubio, 2018), with the consequent more sustainable environmental management. However, it is a highly complicated task since, in addition to the structural complexity of nitrogenase, there are still aspects of its regulation, bioenergetics and interaction with other elements that should be elucidated. Omics sciences allow us to see beyond isolated processes, being able to establish correlations between metabolic changes that are not directly related (Roldán et al., 2021). For this reason, in the present work, biological nitrogen fixation and its relationship with phosphorus mobilization have been studied in a model organism, *A. chroococcum* NCIMB 8003. First, the BNF and the PM proteomes were defined. Subsequently, both processes have been studied simultaneously (FP) and after a metabolic adaptation to phosphorus limitation (FPb), which caused a stop in BNF and the release of ammonia into the extracellular medium (Supplementary Figure S2).

The comparison between the control and the BNF conditions revealed that the establishment of BNF correlates with the induction of proteins related to other proteins and processes (Supplementary Dataset S3), such as phosphonate (A0A0C4WHR7, Achr_17570) and molybdenum transport (A0A0C4WRH0, Achr_39250), cell mobility (PilJ, A0A0C4WWB6, Achr_38300; PilG, A0A0C4WU08, Achr_38330), respiration (A0A0C4WN63, Achr_25030), cofactor and heme synthesis (UroD, A0A0C4WJM3, Achr_6700), and oxidative stress response (superoxide dismutase, A0A0C4WN89, Achr_25450; glutathione *S*-transferase, A0A0C4WJL1, Achr_5670).

Regarding phosphorus mobilization, it was found that, beyond the necessary metabolic flux compensations, the main enzymes involved in this process are the PhoX domain-containing protein (A0A0C4WPA0), the exopolyphosphatase Achr_36790 (A0A0C4WTQ1) and the extracellular alkaline phosphatase Achr_30330 (A0A0C4WPA5), which could be good candidates to implement these processes in other organisms by synthetic biology approaches (Roldán et al., 2021).

When both processes take place at the same time (BNF and PM compared to C), changes in metabolic pathways were also observed. Thus, the synthesis of pyrimidines (orotidine 5′-phosphate decarboxylase, A0A0C4WNN0, Achr_27610; aspartate carbamoyltransferase, A0A0C4WRA0, Achr_38390) and purines (amidophosphoribosyltransferase, A0A0C4WRC5, Achr_14230; phosphoribosylamine-glycine ligase, A0A0C4WVR5, Achr_34370), as well as *L*-methionine (*O*-succinylhomoserine sulfhydrylase, A0A0C4WHE2, Achr_14240; aspartate-semialdehyde dehydrogenase, A0A0C4WRB7, Achr_14130), was strongly induced in the FP condition. In addition, in the FPb condition, a connection with copper was also observed since the multicopper oxidase type 2 (A0A0C4WIZ7, Achr_2720) was induced almost 500 times. The expression of enzymes related to oxidative stress (such as AhpC, A0A0C4WU67, Achr_39540, induced 138 times; or cytochrome *c* catalase A0A0C4WQI9, Achr_35330, 81-fold induced) was also enhanced. On the other hand, down-regulated proteins were methionine synthase (vitamin-B_12_ independent) isozyme (A0A0C4WUU0, Achr_28820), biotin synthase (A0A0C4WJG1, Achr_5850), and alginate biosynthesis proteins (Alg44, A0A0C4WPI8, Achr_3120).

When BNF was compared to PM (BNF vs. PM and BNF vs. FP), GO categories related to biosynthesis of alginic acid, polyhydroxybutyrate and acetyl-CoA, as well as iron transport, were up-represented among proteins induced when phosphorus mobilization was active (Supplementary Dataset S3). On the other hand, the synthesis of protoporphyrinogen IX was related to BNF since it was up-regulated in BNF and FP compared to C, PM and FPb. In fact, the uroporphyrinogen decarboxylase (A0A0C4WJM3, Achr_6700) showed a fold-change ≈ 3 in the comparisons BNF vs. C, BNF vs. PM and FP vs. PM; and the coproporphyrinogen-III oxidase (A0A0C4WN44, Achr_25010) also showed an increment in BNF, but not in FP (Supplementary Dataset S3). The reduction of nitrogen fixation in FPb was corroborated when comparing BNF vs. FPb and FP vs. FPb. It was also observed that in FPb, when compared to BNF and FP, the transport of iron and molybdenum, as well as the response to oxidative stress, were over-represented. Likewise, in BNF and FP compared to FPb, the metabolism of glycoxylate, acetyl-CoA, acetate and propionate was up-regulated whereas cobalamin biosynthesis, cell redox processes and iron–sulfur cluster assembly were down-represented (Supplementary Dataset S3).

## 4. Conclusion

The main conclusions of this work are: (1) in *A. chroococcum* NCIMB 8003, BNF requires important metabolic changes beyond the genes directly related to nitrogen fixation, such as the synthesis of heme group, “*de novo*” synthesis of *L*-methionine and other processes; (2) the main phosphatases responsible for P mobilization that were detected by proteomics are the PhoX domain-containing protein (A0A0C4WPA0) and the exopolyphosphatase Achr_36790; (3) BNF and PM are closely related, so that phosphorus limitation stops BNF, although BNF can potentiate PM; and (4) both BNF and PM influence alginate synthesis in this strain.

## Data availability statement

The datasets presented in this study can be found in online repositories. The names of the repository/repositories and accession number(s) can be found in the article/Supplementary material.

## Author contributions

KB, CL, and GR-C: investigation, methodology, and data curation. FL-T and JH-C: investigation and methodology. PC, LS, VL-A: methodology and data curation. MR: conceptualization, supervision, and funding acquisition. CM-V: conceptualization, supervision, funding acquisition, and project administration. AO-A: software, conceptualization, supervision, writing—review and editing, funding acquisition, and project administration. All authors contributed to the article and approved the submitted version.

## Funding

This work was funded by the Plan Propio-2019 of the University of Córdoba, Spain.

## Conflict of interest

The authors declare that the research was conducted in the absence of any commercial or financial relationships that could be construed as a potential conflict of interest.

## Publisher’s note

All claims expressed in this article are solely those of the authors and do not necessarily represent those of their affiliated organizations, or those of the publisher, the editors and the reviewers. Any product that may be evaluated in this article, or claim that may be made by its manufacturer, is not guaranteed or endorsed by the publisher.

## Footnotes

^1^ https://maxquant.org/perseus/

^2^ http://proteomecentral.proteomexchange.org
