# Supplementary material for: Holistic view of biological nitrogen fixation and phosphorus mobilization in Azotobacter chroococcum NCIMB 8003
Source: Front Microbiol. 2023 Feb 8;14:1129721. doi: 10.3389/fmicb.2023.1129721 (PMC9945222; doi:10.3389/fmicb.2023.1129721)
Supplement: Supplementary file 4 [file Table_1.docx]

Table S1. Proteomic search and quantification parameters.

| **Parameter** | | | **Data** |
| --- | --- | --- | --- |
| Specie | *Azotobacter chroococcum* (strain NCIMB 8003) | | **UP000068210** |
| Group specific parameters | Type |  | **Standard** |
|  | Modifications | Variable modifications | **Oxidation (M), Acetyl (Protein N-term)** |
|  |  | Fixed modifications | **Carbamidomethyl ©** |
|  | UHPLC system | Model | **Dionex Ultimate 3000 nano UHPLC system** |
|  |  | Separation column | **Acclaim pepmap C18, 500 nm x 0.075 mm, 2 μm pore size** |
|  |  | Trapping | **0.2 μg/μL at 10 μL/min flow rate, 5 min** |
|  |  | Mobile phase | **2% acetonitrile / 0.05% trifluoroacetic acid** |
|  |  | Gradient time | **60 min** |
|  |  | Gradient | **5-40 % acetonitrile / 0.1 % formic acid** |
|  |  | Flow rate | **300 nL/min** |
|  | Instrument | Max. number of modifications per peptide | **3** |
|  |  | Instrument type | **Orbitrap Fusion** |
|  |  | First search peptide tolerance | **20** |
|  |  | Main search peptide tolerance | **4.5** |
|  |  | Peptide tolerance unit | **ppm** |
|  |  | Individual peptide tolerance | **yes** |
|  |  | Isotope match tolerance | **2** |
|  |  | Isotope match tolerance unit | **ppm** |
|  |  | Centroid match tolerance | **8** |
|  |  | Centroid match tolerance unit | **ppm** |
|  |  | Centroid half width | **35** |
|  |  | Centroid half width unit | **ppm** |
|  |  | Time villey factor | **1.4** |
|  |  | Isotope valley factor | **1.2** |
|  |  | Isotope time correlation | **0.6** |
|  |  | Theoretical isotope corretaliton | **0.6** |
|  |  | Recalibration unit | **ppm** |
|  |  | Min. peak lenght | **2** |
|  |  | Min. DIA peak lenght | **1** |
|  |  | Max. change | **7** |
|  |  | Min score for recalibration | **70** |
|  |  | Cut peaks | **yes** |
|  |  | Gap scans | **1** |
|  |  | Intensity threshold MS1 | **no** |
|  |  | Intensity threshold MS2 | **no** |
|  |  | Check mass deficit | **yes** |
|  |  | Intensity determination | **Value at maximum** |
|  |  | Centroid position | **Gaussian** |
|  |  | DIA initial precursor mass tolerance (ppm) | **20** |
|  |  | DIA initial fragment mass tolerance (ppm) | **20** |
|  |  | DIA corr. threshold for feature clustering | **0.85** |
|  |  | DIA prec. mass. toll.for feat. clustering (ppm) | **2** |
|  |  | DIA frag. mass. toll.for feat. clustering (ppm) | **2** |
|  |  | DIA score N | **7** |
|  |  | DIA min. score | **1.99** |
|  |  | DIA quant method | **Mixed LFQ split** |
|  |  | DIA feature quant method | **Sum** |
|  |  | DIA top N fragments for quant | **10** |
|  |  | DIA top msms intensity quantile for quant | **0.85** |
|  |  | DIA min. Msms intensity for quant | **0** |
|  |  | DIA precursor filter type | **None** |
|  |  | DIA min. fragment overlap score | **1** |
|  |  | DIA min. precursor score | **0.5** |
|  |  | DIA min. profile correlation | **0** |
|  |  | DIA global ML | **yes** |
|  |  | DIA adaptative mass accuracy | **no** |
|  |  | DIA mass window factor | **3.3** |
|  |  | DIA background subtraction | **no** |
|  |  | DIA background subtraction quantile | **0.5** |
|  |  | DIA background subtraction factor | **4** |
|  |  | DIA transfer q-value | **0.3** |
|  |  | DIA LFQ weighted median | **no** |
|  |  | DIA XGBoost Base Score | **0.4** |
|  |  | DIA XGBoost Sub Sample | **0.9** |
|  |  | DIA XGBoost learning objective | **Binary logistic raw** |
|  |  | DIA XGBoost Min child weight | **9** |
|  |  | DIA XGBoost Maximum Tree Depth | **12** |
|  |  | DIA XGBoost Estimators | **580** |
|  |  | DIA XGBoost Gamma | **0.9** |
|  |  | DIA XGBoost Max Delta Step | **3** |
|  |  | DIA no ML | **no** |
|  |  | DIA only isos for recalibration | **yes** |
|  |  | DIA min. peaks for recalibration | **5** |
|  | Digestion | Digestion mode | **Specific** |
|  |  | Enzyme | **Trypsin** |
|  |  | Max. missed | **2** |
|  |  | Incubation time | **Overnight** |
|  | Label-free quantification | LFQ |  |
| Global parameters | Sequences | FASTA file | ***Azotobacter chroococcum* NCIMB 8003** |
|  |  | Uniprot | **UP000068210** |
|  |  | Include contaminants | **yes** |
|  |  | Min. peptide lenght | **7** |
|  |  | Max. peptide mass (Da) | **4600** |
|  |  | Min. Peptide lenght for unspecific search | **8** |
|  |  | Max. Peptide lenght for unspecific search | **25** |
|  | Protein quantification | Label min. ratio count | **2** |
|  |  | Peptides for quantification | **Unique + razor** |
|  |  | Modifications used in protein quantification | **Oxidation (M), Acetyl (Protein N-term)** |
|  |  | Discard unmodified counterpart peptides | **yes** |
|  |  | Advanced ratio estimation | **yes** |
|  | Identification | PSM FDR | **0.01** |
|  |  | Protein FDR | **0.01** |
|  |  | Site decoy fraction | **0.01** |
|  |  | Min. peptides | **1** |
|  |  | Min. razor + unique peptides | **1** |
|  |  | Min. unique peptides | **0** |
|  |  | Min. Score for unmodified peptides | **0** |
|  |  | Min. score for modificed peptides | **40** |
|  |  | Min. delta score for unmodified peptides | **0** |
|  |  | Min. delta score for modified peptides | **6** |
|  |  | Main search max. combinations | **200** |
|  |  | Razor protein FDR | **yes** |
|  |  | Second peptides | **yes** |
|  |  | Match between runs | **yes** |
|  | Label free quantification | Stabilize large LFQ ratios | **yes** |
|  |  | Require MS/MS for LFQ comparisons | **yes** |
|  |  | Advanced site intensities | **yes** |
|  |  | Top3 | **yes** |
| General parameters | Survey scans of peptide precursors | | **From 400 to 1500 m/z** |
|  | Resolution | | **120 K (at 200 m/z)** |
|  | Ion count target | | **5 × 10^5^** |
|  | Isolation | | **1.6 Da with the quadrupole** |
|  | Fragmentation | | **CID** |
|  | Normalized collision energy | | **35** |
|  | Rapid scan MS | | **yes** |
|  | Samples for MS2 | | **Precursors with 2-5 charge state** |
|  | Run mode | | **Top speed, with 3 s cycles** |
|  | MS2 spectra search | | **MaxQuant software v1.5.7.4** |
|  | Differential expression analysis | | **Perseus software v1.6.12.1** |
|  | Criteria for protein identification | | **At least 2 peptides** |
|  | Criteria for exclusively expressed proteins | | **Present only and, at least, in 2 replicates of one condition** |
|  | Replicates | | **3** |
|  | Conditions | | **5 (C, BNF, PM, FP, FPb)** |
|  | Differential expression if | | **Fold change ≥ 2 and *p* value < 0.05 (Benjamini-Hochberg corrected)** |
