# Supplementary material for: Holistic view of biological nitrogen fixation and phosphorus mobilization in Azotobacter chroococcum NCIMB 8003
Source: Front Microbiol. 2023 Feb 8;14:1129721. doi: 10.3389/fmicb.2023.1129721 (PMC9945222; doi:10.3389/fmicb.2023.1129721)
Supplement: Supplementary file 5 [file Table_2.docx]

Table S2. Primer used in this work for qRT-PCR analysis.

| Achr_39420 | nifL | Nitrogen fixation regulatory protein, NifL | AGCAGGCCCACGTGTTCTTCACCCC | CCTCCTCGGCCATCAGCGCCTTCA |
| --- | --- | --- | --- | --- |
| Achr_39430 | nifA | Nif-specific sigma54-dependent transcriptional activator protein, NifA | CCTGCTGCACGGCCTGGTGACCAT | TTGCCGATCACGCCTTCGCCGCTT |
| Achr_1270 | nifD | Nitrogenase protein alpha chain (EC 1.18.6.1) | TCTCCATCCAGTCCGAGTGCCCGAT | AACGCCACGGAAGCCTTCGCAAC |
| Achr_6220 |  | Alkaline phosphatase, Location: Unknown | AGCTGGATCCCTCGGTCCGCGTCT | GGCTCACCCAGGCCGTCCGCGAAT |
| Achr_19430 | nudJ | Phosphatase NudJ (EC 3.6.1.-) | ATTGGCAGGCACACGTCACCGT | ACTGTACGGCCATCCTGTAGCTCCT |
| Achr_30330 |  | Alkaline phosphatase D | CCCCAACCCGCTGGACGGCACCT | CGACACCCGCAGCAGGCCGCTCT |
| Achr_3580 | phoR | Phosphate regulon sensor kinase PhoR | AGCAGCGCCTTTTCCAACCTGGTGT | GTCCCGGTCGCCCCACCAGCGGAT |
| Achr_33070 |  | Acid phosphatase/vanadium-dependent haloperoxidase superfamily | GGCGTGCACTGGCCGACCGACATCA | CAGCGCCAGGCACAGCGGCACGA |
| Achr_35150 | rpoB | DNA-directed RNA polymerase subunit beta | CCGTCGCCGCTTCGCTGATTCCC | ACGTTGCGCTCCATCCCGGTACCC |
