# Supplementary material for: Holistic view of biological nitrogen fixation and phosphorus mobilization in Azotobacter chroococcum NCIMB 8003
Source: Front Microbiol. 2023 Feb 8;14:1129721. doi: 10.3389/fmicb.2023.1129721 (PMC9945222; doi:10.3389/fmicb.2023.1129721)
Supplement: Supplementary file 6 [file Table_3.docx]

Table S3. Collecting point conditions.

| **Sample** | **CFU (10^8^)** | **[NH_4_^+^] (mM)** | **[P] (mg/L)** | **pH** |
| --- | --- | --- | --- | --- |
| **C** | 6.5±0.46 | 3.61±0.54 | 551.7±28.41 | 6.95±0.01 |
| **BNF** | 6.7±0.1 | 0.06±0.01 | 385.51±29.84 | 7.13±0.01 |
| **PM** | 4.6±0.65 | 1.17±1.99 | 26.56±1.99 | 6.97±0.01 |
| **FP** | 6.4±0.69 | 0.02±0.01 | 29.57±4.58 | 7.01±0.01 |
| **FPb** | 10.3±1.1 | 0.07±0.01 | 114.74±2.39 | 6.44±0.01 |
