# Supplementary figures and images for: Holistic view of biological nitrogen fixation and phosphorus mobilization in Azotobacter chroococcum NCIMB 8003
Source: Front Microbiol. 2023 Feb 8;14:1129721. doi: 10.3389/fmicb.2023.1129721 (PMC9945222; doi:10.3389/fmicb.2023.1129721)

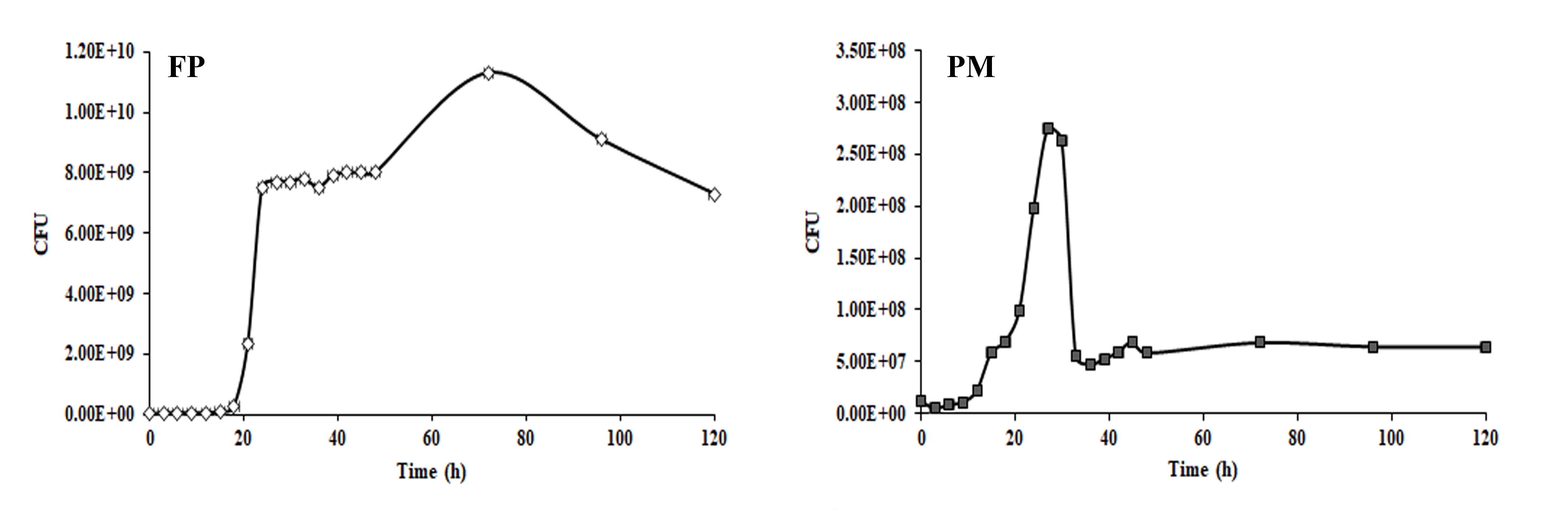

Supplement: Supplementary file 7 [file Image_1.TIF]

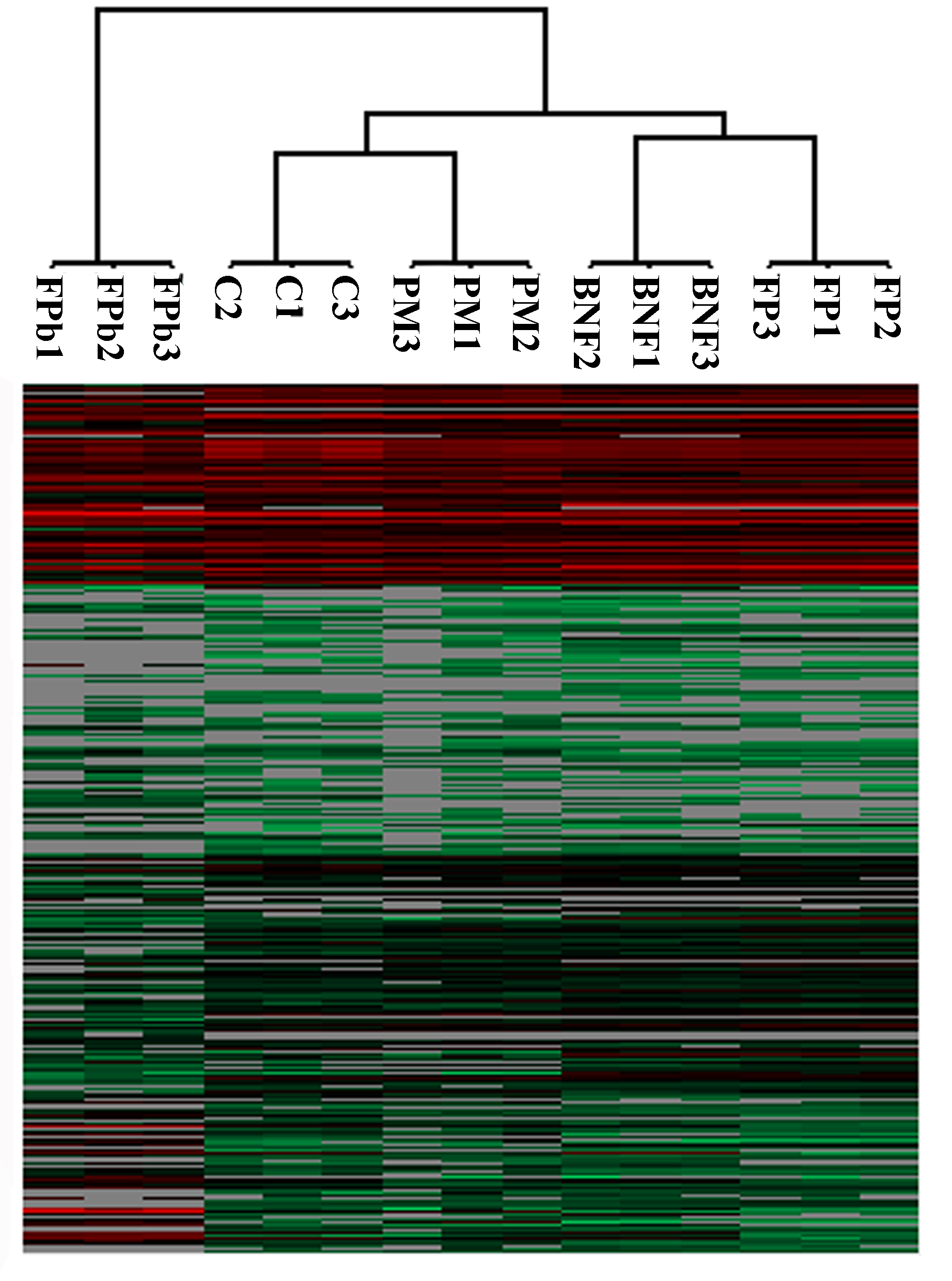

Supplement: Supplementary file 8 [file Image_2.TIF]

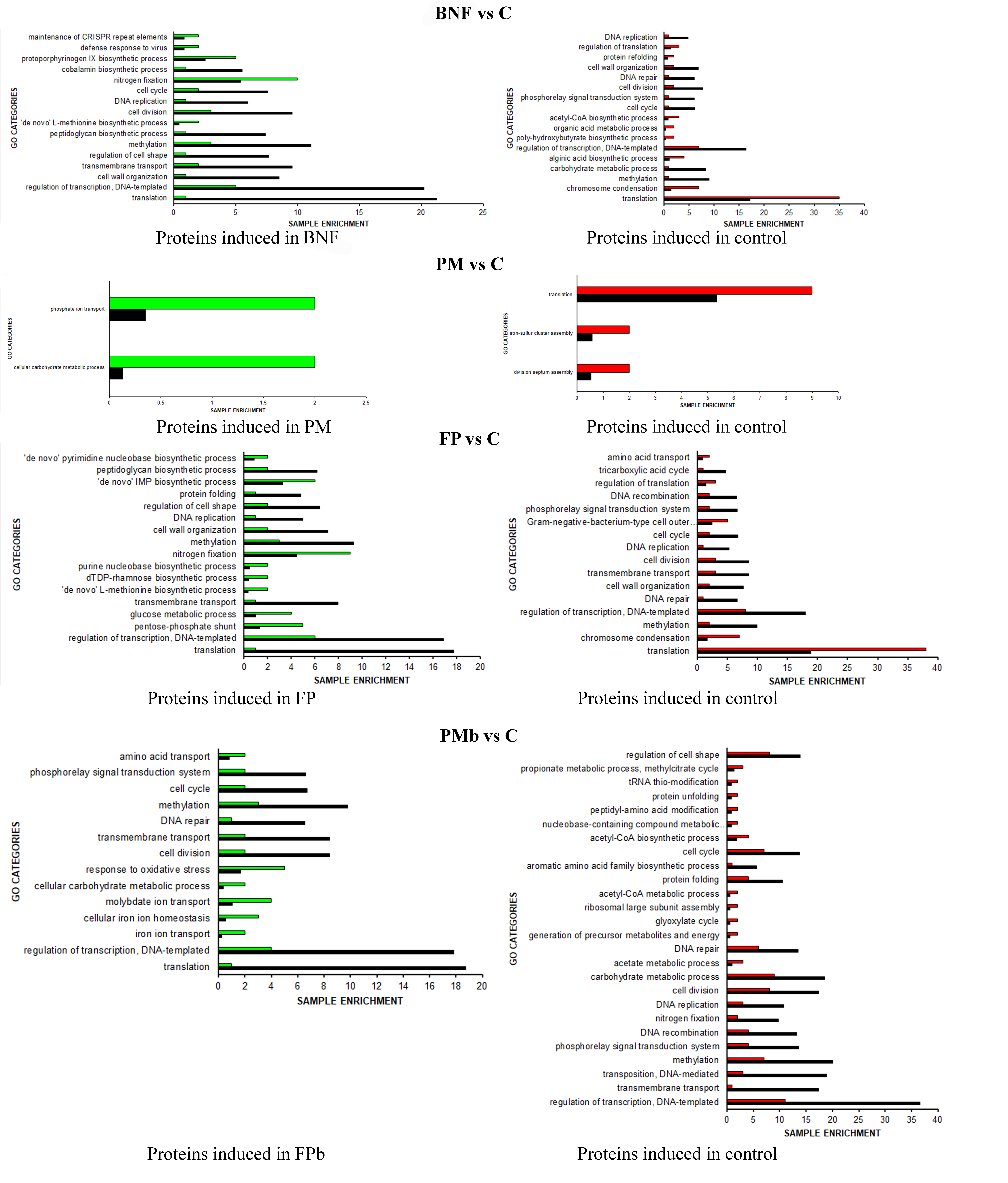

Supplement: Supplementary file 9 [file Image_3.TIF]

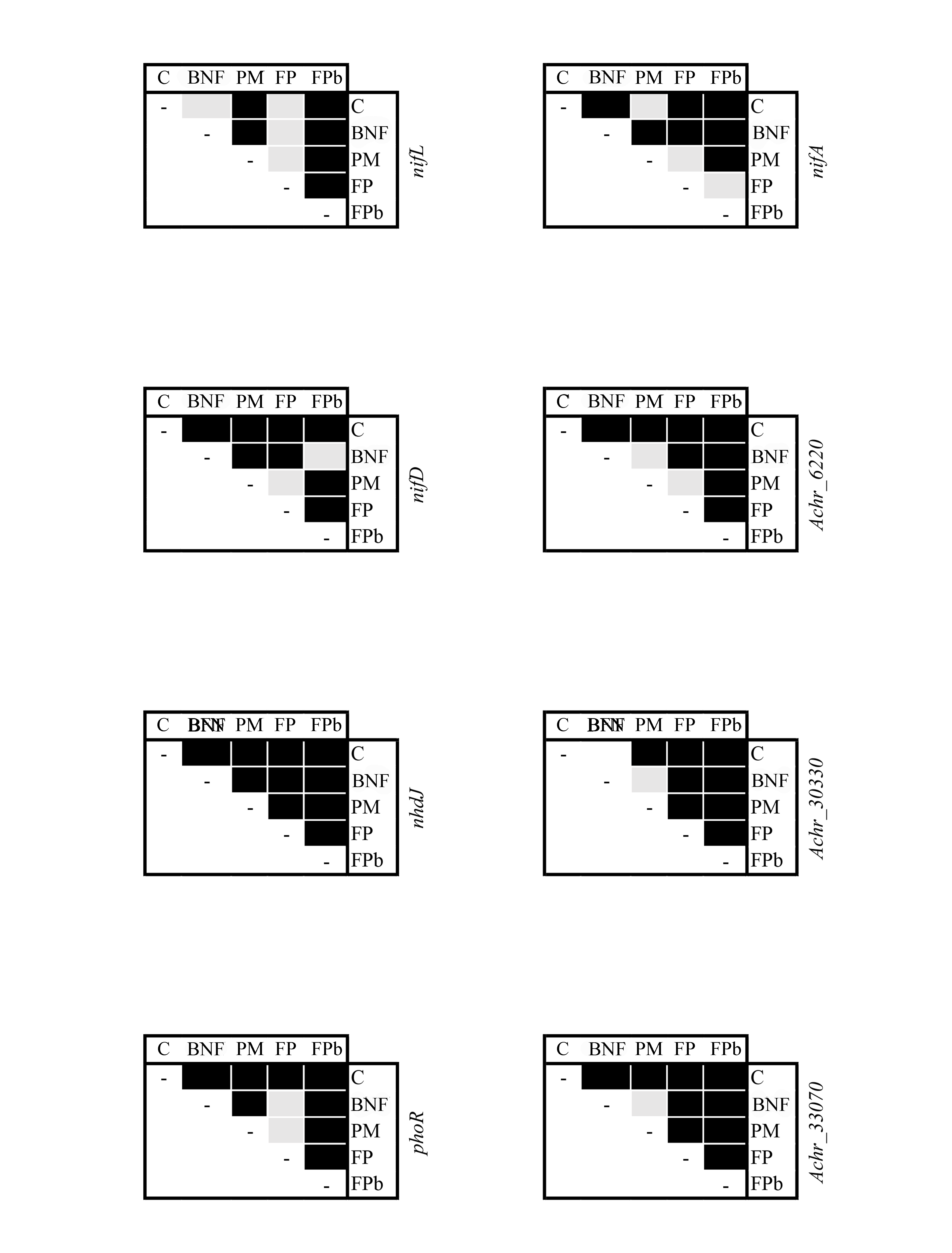

Supplement: Supplementary file 10 [file Image_4.TIF]
